# The Incidence of Residual Neuromuscular Block in Pediatrics: A Prospective, Pragmatic, Multi-institutional Cohort Study

**DOI:** 10.7759/cureus.56408

**Published:** 2024-03-18

**Authors:** Debra J Faulk, Joelle B Karlik, Kim M Strupp, Stephanie M Tran, Mark Twite, Sorin J Brull, Myron Yaster, Thomas M Austin

**Affiliations:** 1 Anesthesiology, Children's Hospital Colorado, Denver, USA; 2 Anesthesiology, Emory University School of Medicine, Atlanta, USA; 3 Anesthesiology and Perioperative Medicine, Mayo Clinic College of Medicine and Science, Jacksonville, USA; 4 Anesthesiology, University of Florida, Gainesville, FL, USA

**Keywords:** pediatric anesthesia, patient health safety, residual neuromuscular block, postoperative complications, pediatrics, neuromuscular monitoring

## Abstract

Introduction

Residual neuromuscular block, defined as a quantitatively measured train-of-four ratio (TOFr) <0.9, is common postoperatively. Using a pragmatic trial design, we hypothesized that qualitative and/or clinical assessment of neuromuscular block would inadequately detect residual block following antagonism with neostigmine or sugammadex.

Method

After IRB approval and written informed consent, 74 children (aged 2-17 years), undergoing elective surgery and receiving rocuronium, were prospectively enrolled in the study at Children's Hospital Colorado and Children's Healthcare of Atlanta. Routine clinical practice at both institutions consisted of clinical signs and/or qualitative assessment with peripheral nerve stimulators. Children at the Colorado hospital routinely received sugammadex antagonism; whereas children at the Atlanta hospital received neostigmine. Residual neuromuscular block was assessed postoperatively using quantitative electromyography. If TOFr was <0.9, patients received sugammadex until TOFr ≥0.9.

Result

Qualitative and clinical assessment failed to detect residual block in 29.7% of patients in the neostigmine reversal cohort (adjusted odds ratio (aOR) 29.8, 95% confidence interval (CI): 2.7 to 5,559.5, p-value = 0.002). No residual block was detected in the sugammadex reversal cohort. A correlation between increasing patient weight and incidence of postoperative residual block was observed in the neostigmine cohort (aOR 1.05, 95% CI: 1.02 to 1.10, p-value = 0.002).

Conclusion

Qualitative and/or clinical assessment of neuromuscular block inadequately detects residual block following neostigmine antagonism.

## Introduction

Neuromuscular blocking agents, such as rocuronium or vecuronium, are used routinely by anesthesiologists during adult and pediatric surgery to create optimal conditions for tracheal intubation, surgical exposure, and intraoperative controlled mechanical ventilation. Evaluating the return of adequate neuromuscular function using clinical tests (e.g., a 5-sec head lift, adequate tidal volume) or qualitative peripheral nerve stimulation before extubation is common but notoriously inaccurate, often resulting in a residual neuromuscular block in the postanesthesia care unit (PACU) [1-5]. The introduction of sugammadex into American medical practice in 2015 reduced the incidence of residual neuromuscular block, defined as a quantitatively measured train-of-four ratio (TOFr) <0.9, but did not eliminate it [6,7]. In children, the incidence of residual neuromuscular block is similar to that in adults and may be as high as 50% [8].

Only quantitative assessment of neuromuscular function with electromyography (EMG), mechanomyography, or acceleromyography can accurately measure a TOFr [2,3]. Unfortunately, user-friendly, quantitative monitors have not been readily available in clinical practice, particularly in pediatrics. Indeed, the most common quantitative monitors in clinical use are acceleromyography-based devices that do not work well in children because they require a time-consuming stabilization period, calibration, a baseline measurement for normalization, and most importantly, they require a free, untucked arm for accurate evaluation of the evoked thumb movement [9]. On the other hand, EMG-based monitors measure electrical activity (compound muscle action potentials) in response to nerve stimulation as opposed to muscle movement, as occurs in acceleromyography; EMG monitors do not require stabilization, normalization, or an untucked arm [2,3,9]. Recently, several portable and easy-to-use quantitative EMG monitors have become commercially available (TwitchView®, Blink Device Company, Seattle, WA; TetraGraph, Senzime AB, Uppsala, Sweden).

Using a pragmatic trial design [10,11], we hypothesized that qualitative and/or clinical assessment of neuromuscular block would inadequately detect minimal levels of a block (train-of-four ratio 0.4-0.9) following antagonism with either neostigmine or sugammadex in pediatric patients.

## Materials and methods

After obtaining Investigational Review Board (IRB) approval, written parental informed consent, and when appropriate, patient assent, we conducted a prospective, observational pragmatic trial in two institutions, Children's Hospital Colorado (CHCO) and Children's Healthcare of Atlanta (CHOA), to evaluate the incidence of residual neuromuscular block in children in routine pediatric anesthesia practice. American Society of Anesthesiologists (ASA) physical status 1-3 patients, between two and 17 years of age undergoing elective surgery with the planned use of rocuronium were enrolled. Exclusion criteria included: neuromuscular disease that may interfere with monitoring, difficult airway, total intravenous anesthesia (TIVA), planned post-operative mechanical ventilation or intensive care unit admission, severe hemodynamic or acid-base disturbances, significant impairment of liver or kidney function, history of emergence delirium, omission of rocuronium from the anesthetic plan, conditions preventing EMG sensor placement, and emergency or after-hours surgery.

At both institutions, routine clinical practice was followed. This involved qualitative assessment of neuromuscular block using peripheral nerve stimulators and/or clinical signs (e.g., attempted spontaneous respirations, adequacy of tidal volumes and/or ventilation while still anesthetized, purposeful or spontaneous displays of strength such as head/leg lift/grabbing for the endotracheal tube, adequate tidal volume/respiratory rate, etc.) during emergence from anesthesia. Thirty-seven (50%) of children at CHCO routinely received sugammadex [mean dose 2.5 mg.kg-1, interquartile range (IQR) 2.0, 4.0], while 37 (50%) of children at CHOA routinely received neostigmine (mean dose 0.05 mg.kg-1, IQR 0.04, 0.07) plus glycopyrrolate for antagonism of neuromuscular block, according to standard institutional and individual clinician practice. Within twenty minutes of arrival in the PACU, recovery from neuromuscular block was assessed via quantitative EMG monitoring (TwitchView®) by study investigators. As our patients were awake during their assessments, we utilized a sub-maximal current setting to evaluate TOFr to minimize discomfort. This was shown to reliably assess patients for residual neuromuscular weakness [12]. Additionally, the TOFr remains constant at any current that is at least 10 mA above the threshold current. In our study, we began with the lowest stimulus current (10 mA) and increased the stimulating current sequentially by 10 mA until an adequate EMG signal was obtained. The current stimulus for testing ranged from 20-40 mA. There were no cases of failure to adequately obtain a TOFr in the PACU.

To minimize any potential Hawthorne effect, attending anesthesiologists at both institutions were told that the purpose of the study was to evaluate the monitors for purchase rather than as an assessment of the adequacy of antagonism in response to any inquiries. In the PACU, if the TOFr was > 0.9 the EMG array was removed. If the TOFr was <0.9, sugammadex was administered by a study investigator, regardless of the intraoperative antagonist received, using a rescue drug dose that was based on the level of neuromuscular block (complete, deep, moderate, shallow, or minimal block) tested every five mins until the TOFr was ≥ 0.9 (Table 1).

**Table 1:** Residual neuromuscular block rescue algorithm with sugammadex doses to be administered in response to the level of residual block assessed in the pediatric postanesthesia care unit. TOFr: train-of-four ratio; PTC: post-tetanic count; TOFc: train-of-four count.

Level of Block	TOFr	Sugammadex Dose	Re-check TOFr
Complete Block	0 PTC	16 mg⋅kg^-1^	5 min
Deep Block	PTC ≥ 1, TOFc = 0	4 mg⋅kg^-1^	5 min
Moderate Block	TOFc = 1-3	2 mg⋅kg^-1^	5 min
Shallow Block	TOFr <0.4	2 mg⋅kg^-1^	5 min
Minimal Block	TOFr = 0.4-0.9	2 mg⋅kg^-1^	5 min
Acceptable Recovery	TOFr ≥ 0.9	N/A	N/A

Finally, we obtained the following data from the electronic medical record: patient demographics, timing, and dosing of rocuronium and antagonist administration, and the presence of documentation of train-of-four count (TOFc) before antagonism. Race was self-reported by the patient’s family.

Statistical analysis

Nominal variables were expressed as count (percentage) while continuous variables were expressed as median IQR. Univariate analyses were conducted utilizing the Wilcoxon Rank Sum test, Fisher’s exact test, or simple Firth’s logistic regression as appropriate for the distributions. Bonferroni’s correction was used to adjust for pairwise comparisons. To limit confounding in this observational analysis, a multivariable Firth’s logistic regression was performed with the rate of residual neuromuscular block in the PACU as the response variable. In addition to the antagonist agent administered (sugammadex vs neostigmine), other explanatory variables included in this model were weight, body mass index (BMI) percentile, race (African American vs. non-African American), time from last rocuronium dose to first dose of antagonist, overall rocuronium dose per hour of surgery (in mg.kg-1.hr-1), and train-of-four (TOF) response checked within 15 min of reversal. Since there was a significant correlation between residual neuromuscular block and weight, a weight cut point was determined that maximized sensitivity and specificity using Youden’s index while a 95% CI for this cut point was determined by bootstrapping. The multivariable regression analysis was then repeated with weight re-parameterized as a binary variable based on this cut point. Lastly, to determine the effect of over-parameterization on parameter estimates and their corresponding CIs, regression models were reconstructed with covariates omitted. All analyses were performed using the R statistical software package (Version 4.1.2, R Foundation, Vienna, Austria) with the ‘logistf’ package used for the regression analysis and ‘cutpointr’ used to establish the weight cut point. All stated p values were two-tailed and p values of <0.05 were considered statistically significant.

Rates of residual neuromuscular block in the adult population are different for the two antagonists (43.4% neostigmine vs 0% sugammadex) [13]. To show a difference in the proportion of 27.2% [exact inner 95% confidence interval bounds (32.1% vs 4.9%)] between the two groups with an alpha error of 5% and a beta error of 10%, 37 patients were needed at each site to power the study appropriately using Fisher’s Exact Test.

## Results

Seventy-four patients were studied, 37 (50%) patients in each cohort. Patient demographics, including age, weight, sex, race, and ASA physical status classification were recorded for all patients (Table 2).

**Table 2:** Patient demographics for the sugammadex and neostigmine patient cohorts. Values for nominal variables are count (percentage) while continuous variables are median (interquartile range, IQR). *Pairwise P-value; CHCO: Children’s Hospital of Colorado; CHOA: Children’s Hospital of Atlanta; BMI: body mass index; ASA: American Society of Anesthesiologists.

	CHCO/Sugammadex (n = 37)	CHOA/Neostigmine (n = 37)	Overall and *Pairwise P-value
Sex (M/F)	24 (64.9%) / 13 (35.1%)	18 (48.6%) / 19 (51.4%)	0.24
Age (years)	13 (IQR 6, 16)	12 (IQR 8, 15)	0.57
Weight (kg)	42.4 (IQR 22.4, 62.1)	48.7 (IQR 24.4, 83.0)	0.27
BMI (kg⋅m^-2^)	19.5 (IQR 16.5, 22.7)	21.1 (IQR 16.0, 32.4)	0.19
BMI (percentile)	73 (IQR 28, 89)	82 (IQR 36, 99)	0.18
ASA Classification			0.26
1	11 (29.7%)	6 (16.2%)	
2	18 (48.6%)	18 (48.6%)	
3	8 (21.6%)	13 (35.1%)	
Race/Ethnicity			<0.001
White	17 (45.9%)	17 (45.9%)	*1.0
African American	2 (5.4%)	14 (37.8%)	*0.008
Hispanic	7 (18.9%)	5 (13.5%)	*1.0
Other	8 (21.6%)	1 (2.7%)	*0.17
Unknown	3 (8.1%)	0 (0.0%)	*1.0

Demographic data in the two cohorts were similar for age (13 vs. 12 yr), weight (42 vs 49 kg), and ASA physical status classification (46% of patients in the two groups were ASA physical status 2), but a significant difference existed in patients’ race/ethnicity; the proportion of African American children at CHOA (37.8%) was significantly greater than the proportion of patients at CHCO (5.4%) (pairwise p-value = 0.008, Table 2). Patients in the CHCO cohort who received sugammadex for antagonism of the neuromuscular block had significantly longer surgeries than the patients at CHOA (125 min vs. 64 min) and received higher total doses of rocuronium, likely due to the longer surgical duration at CHCO (125 min vs. 64 min) (Table 3).

**Table 3:** Perioperative variables for patients receiving sugammadex or neostigmine for antagonism of neuromuscular block. Nominal variables are count (percentage) while continuous variables are median IQR. CHCO: Children’s Hospital of Colorado; CHOA: Children’s Hospital of Atlanta; IQR: interquartile range; TOF: Train of Four; NM: Neuromuscular; PACU: Postoperative Care Unit.

	CHCO/Sugammadex (n = 37)	CHOA/Neostigmine (n = 37)	P-value
Surgical Duration (min)	125 (IQR 55, 203)	64 (IQR 32, 104)	0.003
Total Rocuronium Doses	2 (IQR 1, 4)	1 (IQR 1, 2)	0.19
Total Rocuronium Administered (mg⋅kg^-1^)	1.1 (IQR 0.9, 1.9)	0.6 (IQR 0.5, 0.8)	<0.001
Rocuronium Administered per Surgical Hour (mg⋅kg^-1^⋅hr^-1^)	0.60 (IQR 0.46, 1.09)	0.62 (IQR 0.42, 1.07)	0.75
Time Between Last Dose of Rocuronium and Reversal (min)	76 (IQR 54, 102)	46 (IQR 34, 78)	0.02
Sugammadex Dose (mg⋅kg^-1^)	2.5 (IQR 2.0, 4.0)	---	
Neostigmine Dose (mg⋅kg^-1^)	---	0.05 (IQR 0.04, 0.07)	
Reversal Redose (Yes)	1 (2.7%)	1 (2.7%)	1.0
Number of TOF Checked Intraoperatively	1 (IQR 0, 2)	2 (IQR 1, 4)	<0.001
TOF Checked Within 15 Minutes of Reversal (Yes)	12 (32.4%)	24 (64.9%)	0.010
Residual NM Block in PACU	0 (0.0%)	11 (29.7%)	<0.001

However, when adjusted for surgical duration, the hourly rocuronium doses (0.60 mg⋅kg^-1^⋅hr^-1^ at CHCO and 0.62 mg⋅kg^-1^⋅hr^-1^ at CHOA) were similar between the two cohorts (Table 3). The two cohorts also differed in time between the last dose of the neuromuscular blocking agent and the first dose of the reversal agent, which was significantly longer in the CHCO cohort (76 min) than at CHOA (46 min) (Table 3); the cohorts also differed in the number of times TOF was checked intraoperatively (once at CHCO vs. twice at CHOA, p-value < 0.001, Table 3). Neuromuscular TOF responses within 15 min of administration of the neuromuscular antagonist were assessed more frequently (64.9% of patients) at CHOA than at CHCO (32.4% of patients) (Table 3). There were no differences between institutions regarding end-tidal sevoflurane concentrations or patient body temperature (36.3 oC vs. 36.4 oC, p-value = 0.43) at the time of reversal.

There was a significantly higher incidence of residual neuromuscular block in the neostigmine (CHOA) cohort (29.7% vs. 0.0%, p-value <0.001) relative to the sugammadex (CHCO) cohort. This significant difference persisted after multivariable adjustment [adjusted odds ratio (OR) 29.8] (Table 4).

**Table 4:** Predictors of Residual Neuromuscular Block in patients receiving neostigmine for antagonism of neuromuscular block. Values are odds ratio and 95% confidence intervals (CI). BMI: body mass index; TOF: train of four.

Characteristic	Odds Ratio	95% CI	P-value
Neostigmine Antagonism (vs. Sugammadex)	29.8	2.7 to 5559.5	0.002
Weight (kg)	1.05	1.02 to 1.10	0.002
BMI (percentile)	0.99	0.95 to 1.02	0.29
African American Race (vs. Non-African Americans)	1.14	0.16 to 7.81	0.38
Rocuronium Administered per Surgical Hour (mg⋅kg^-1^⋅hr^-1^)	1.97	0.50 to 7.90	0.45
Time Between Last Dose of Rocuronium and Reversal (min)	0.97	0.92 to 1.01	0.36
TOF Checked Within 15 Minutes of Reversal (Yes vs No)	0.98	0.14 to 6.71	0.83

The median time from neostigmine administration in the operating room until TOF measurement in the PACU was 18 min. In addition, there was a correlation between patient weight and incidence of residual neuromuscular block (Table 4). The cut point for the weight that maximized the Youden’s index for its correlation with a residual neuromuscular block was 83 kg (95% CI: 50 to 111 kg). Thus, patients who weighed 83 kg or more had a higher likelihood of having residual neuromuscular block than those who weighed less than 83 kg (aOR 57.3, 95% CI: 4.54 to 3572.8, p-value < 0.001). There were no significant differences in residual neuromuscular block statistics and CIs after eliminating all covariates from the regression models. Of note, all patients who had residual neuromuscular weakness when tested quantitatively in the PACU had been assessed intraoperatively with subjective (qualitative) tests and were found to have a TOFc = 4 prior to extubation (Table 5).

**Table 5:** Patients whose block was antagonized with neostigmine and experienced residual neuromuscular block all had a train-of-four count of 4 documented prior to extubation and minimal levels of residual neuromuscular block detected by quantitative monitoring with electromyography-based devices in the postanesthesia care unit. The presence or absence of TOF fade prior to or after reversal was not specified ASA: American Society of Anesthesiologists; TOF: train-of-four; TOFr: train-of-four ratio; PACU: postanesthesia care unit

Patient Number	Age	Sex	Weight (kg)	BMI Percentile (%)	ASA Status	Total Rocuronium Dose (mg⋅kg^-1^⋅hr^-1^)	Neostigmine Dose (mg⋅kg^-1^)	Type of Extubation	Qualitative TOF Assessment Prior to Extubation	Initial Quantitative TOFr in PACU
1	12	F	111.1	99	2	0.84	0.05	Deep	4/4	0.68
2	16	F	137.3	99	3	0.43	0.04	Awake	4/4	0.87
3	18	M	163.2	99	2	0.42	0.03	Awake	4/4	0.67
4	15	F	83	98	2	1.11	0.02	Awake	4/4	0.61
5	15	F	51.1	76	2	0.73	0.07	Awake	4/4	0.68
6	15	M	49.7	5	2	0.68	0.07	Awake	4/4	0.85
7	16	M	102.4	99	2	0.20	0.05	Awake	4/4	0.79
8	15	F	90	99	2	0.45	0.06	Awake	4/4	0.60
9	16	F	114	99	3	0.40	0.04	Awake	4/4	0.85
10	2	M	12	2	2	3.33	0.07	Deep	4/4	0.67
11	14	F	118	99	2	0.46	0.04	Awake	4/4	0.81

The subjective assessment of either the presence or absence of TOF fade was not documented.

## Discussion

Despite utilizing qualitative assessments and/or clinical signs for the intraoperative management of neuromuscular block and its antagonism, this prospective, pragmatic trial at two different children’s hospitals that used quantitative monitoring in the PACU revealed a high incidence (29.7%) of residual neuromuscular block after neostigmine antagonism, while this incidence after sugammadex was 0%. This is consistent with previous studies performed in both adults and children [5-8, 13-15]. Our study confirms the importance of quantitative monitoring to accurately determine the depth of the block and that failure to use it and/or reliance on clinical signs results in a residual neuromuscular block after neostigmine reversal in the pediatric PACU [8]. Although we found residual block only in patients who received neostigmine antagonism, our study was not powered to determine if it would also occur following sugammadex antagonism. Additionally, we found that increasing patient weight may increase the risk of residual neuromuscular block when using neostigmine, similar to reports in adults; this association should be confirmed in future pediatric studies. Finally, this pragmatic trial reflects variations in real-life clinical practice among different hospitals and how these variations affect clinical decision-making and patient outcomes.

A pragmatic trial differs from a randomized controlled trial because it assesses data sampled routinely in clinical practice and alters routine care minimally or not at all [10,11]. On the other hand, a randomized controlled trial does not reflect the real world. Rather, key design features of randomized controlled trials, including extensive exclusion criteria and study populations that differ from the general patient population, limit the generalizability of the findings in this type of investigation [10,11]. We chose a pragmatic design to evaluate patients in routine clinical practice and in whom our study results might be practically applied.

As can be seen in our results, in real-world conditions, anesthesiologists tend to overestimate the degree of spontaneous recovery when using qualitative means of assessment of responses from peripheral nerve stimulators [3,14]. These nerve stimulators are not true monitors, but devices that deliver an electrical impulse to the nerve and rely on the provider’s subjective (and often incorrect) interpretation of the resulting muscle response [1,3]. The occurrence of residual block in our neostigmine cohort highlights how notoriously inaccurate our qualitative evaluation can be, demonstrating our tendency to overestimate TOFc and underestimate TOF fade [16]. Despite qualitative assessment in the operating room and a documented TOFc of four obtained with a peripheral nerve stimulator, quantitative monitors revealed that 11 (29.7%) of children receiving neostigmine antagonism experienced residual neuromuscular block in the PACU (a measured TOFr between 0.6 and 0.9). At the minimal levels of block defined in Table 1, patients are in a “blind zone of paralysis” that is undetectable by clinical or subjective evaluation but one that can have real physiologic significance. Diaphragmatic function may be recovered, but genioglossus muscle strength and pharyngeal tone likely are still impaired, even at a TOFr of 0.9 [17,18]. This residual muscle weakness even with minimal block (TOFr = 0.4-0.9) can lead to collapse and obstruction of the pediatric upper airway and risk of aspiration due to uncoordinated swallowing. The hypoxic ventilatory response is also blunted at this incomplete level of recovery, predisposing this vulnerable patient population to hypoxia and hypoventilation [19].

The current “gold” standard threshold for neuromuscular recovery is a quantitative TOFr > 0.9 measured at the adductor pollicis (Table 1). However, in pediatric patients, the arms are routinely tucked under surgical drapes and are inaccessible intraoperatively; in such cases, the default site for monitoring is often the corrugator supercilii muscle, which is well known to overestimate the degree of neuromuscular recovery [3,20]. This overestimation occurs because different muscles respond to and recover from neuromuscular blocking agents at different rates. Peripheral muscles (e.g., adductor pollicis) are more sensitive to neuromuscular blocking agents than central facial muscles (e.g., corrugator supercilii). Facial muscles are also more prone to direct muscle stimulation leading to misinterpretation of greater neuromuscular recovery than actual recovery. A goal of this study was to observe the incidence of a residual neuromuscular block under real-world conditions, so no recommendations were made as to when, where, or how the levels of the neuromuscular block were to be assessed by the anesthesia practitioners. Routine monitoring of facial muscles, as commonly occurs in children despite specific recommendations against this practice [21], may have therefore contributed to the high rates of residual block in the neostigmine group.

The only reliable means of detecting minimal levels of block (TOFr 0.4-0.9) is with the use of quantitative monitors, particularly if neostigmine is used. However, the lack of available devices that are simple and easy to use has contributed to their infrequent adoption into clinical practice [9]. Several modalities have been utilized in the past, including mechanomyography, kinemyography, acceleromyography, and EMG. Mechanomyography has been considered the gold standard, though it has mainly been used for research purposes and is not commercially available. Acceleromyography is the modality used most commonly in clinical practice and several portable, relatively user-friendly monitors are commercially available. This modality measures the acceleration of muscle contraction (preferably at the adductor pollicis) and therefore requires a free, unencumbered hand for use. Monitoring accuracy relies on baseline stabilization of responses, calibration, and normalization; despite these time-consuming procedures, acceleromyography tends to overestimate recovery and a TOFr of 0.95 or 1.0 (rather than 0.9) has been proposed to indicate full recovery with its use [1,4]. The biggest barrier to routine adoption of acceleromyography in children is the need for unencumbered access to a limb. This requirement is likely the major reason for clinicians’ avoidance of using this type of quantitative monitor at one of our two sites (CHOA). Recently, several portable EMG devices have been developed that operate by measuring compound action potentials and therefore do not require unencumbered movement of the monitored muscle (adductor pollicis). Baseline stabilization is unnecessary, and the monitor calibration requires less than a minute [22]. Therefore, these devices may overcome several barriers to neuromuscular monitoring in pediatric patients, supporting adoption into clinical practice. Currently, EMG electrode arrays are commercially available for children over the age of one month. At the time of this study, EMG electrode arrays were only available for children two years of age or greater. Therefore, the neonatal to two-year-old population was not investigated in our study.

While 11 (29.7%) of the children in the neostigmine cohort experienced residual weakness in the PACU, the incidence of residual neuromuscular block in the patients receiving sugammadex (CHCO) was zero. This occurred despite experiencing factors that generally increase the likelihood of developing residual neuromuscular block: patients who received sugammadex had longer surgeries, received greater total doses of neuromuscular blocking agents, and had less frequent subjective assessment of TOFc than patients receiving neostigmine (CHOA). The practice of using sugammadex to supplant the use of quantitative monitoring reflects previously reported practice patterns in pediatric anesthesia, in which sugammadex has become the agent of choice while the use of peripheral nerve stimulators has become less common [23]. Since gaining the United States Food and Drug Administration approval for use in adults in 2015, studies have shown that sugammadex antagonizes neuromuscular block more rapidly, regardless of the depth of block, and has a better safety profile than neostigmine [24-26]. These benefits are supported by our findings at CHCO, where despite the existence of factors that generally increase the incidence of residual neuromuscular block (longer surgical times, greater total dose of neuromuscular blocking agent administration, less intraoperative TOF monitoring, and less TOF testing prior to antagonism), CHCO patients receiving sugammadex did not experience residual neuromuscular block (TOFr <0.90). Nevertheless, both residual block and its recurrence after apparent recovery have been reported in adult and pediatric patients antagonized with sugammadex, especially in the absence of monitoring, and such practice cannot be recommended [6,7]. We found no residual neuromuscular block with the use of sugammadex in this study, likely because it was not powered to investigate this outcome. Therefore, the exclusion of quantitative monitoring from routine anesthesia practice presents significant safety concerns and cannot be recommended solely based on our results at CHCO (Table 3). The results of this pragmatic study confirm that qualitative monitoring cannot ensure appropriate dosing of antagonists and complete return of neuromuscular function, particularly when neostigmine is used.

Finally, our data show a correlation between patient weight and the occurrence of residual neuromuscular block in children, particularly when neostigmine is used (Table 4). We found patients in the neostigmine cohort weighing 83 kg or greater to be at 57 times greater risk of experiencing residual block than those weighing less than 83 kg. Medication dosing in pediatric anesthesia is usually based on the patient’s weight at the time of surgery, or total body weight. However, the suggested dosing for rocuronium is based on ideal body weight. Most patients experiencing residual neuromuscular block in this study received hourly doses of rocuronium that were near or exceeded intubating doses based on total body weight, and the median total body weight of patients experiencing residual block was 2.1 times that of their ideal body weight. Eight of the 11 (73%) patients with residual weakness had a BMI in the 98 or greater percentile (Table 5). Regardless of rocuronium dosing, however, the maximum recommended dose of neostigmine is 5 mg, corresponding to a 0.07 mg⋅kg-1 dose in a 71-kg patient. The ceiling effect of neostigmine may contribute to the occurrence of residual weakness in patients receiving unintentionally excessive doses of neuromuscular blocking agents.

There are several limitations to our study. As in all pragmatic trials, anesthesia care, monitoring, and documentation were not controlled, potentially introducing bias. A potential additional bias was the influence of ethnic differences between the two hospital cohorts. In addition, usual care at the two participating sites naturally divided patients receiving neostigmine (CHOA) and those receiving sugammadex (CHCO) into a single institution. Other study limitations include: the median age of the patients was 12-13 y, which might have limited the scope of the generalization to younger (or very young) children. In addition, the BMI was in the lower range, and this might not reflect issues related to morbidly obese children. Further, most patients were ASA physical status 1-2 and therefore this study may not reflect the outcomes in a sicker population. 

While we cannot exclude that differing culture and practice system influences may have contributed to the comparative rates of the residual neuromuscular block between the neostigmine and sugammadex groups, institutional influences should not impact rates within each cohort, as each was confined to a single institution. The rate of residual neuromuscular block reported for the neostigmine group is therefore credible and consistent with previous reports [8]. It is possible our results were influenced by the Hawthorne effect. Anesthesia providers were not explicitly informed of this study but could have become indirectly aware due to our activities. This may have influenced how, or if, they administered antagonists. Finally, the extremely wide confidence intervals limit the precision of these statistics. However, this is likely due to the relatively small sample size and the zero occurrences of residual neuromuscular block in the sugammadex cohort, rather than issues with our statistical modeling (i.e., lack of convergence).

## Conclusions

This prospective, observational, pragmatic study provides important information on the potential risks facing pediatric patients who receive neuromuscular blocking agents during general anesthesia. Indeed, during routine anesthetic care, there is a significant and unacceptably high incidence of residual neuromuscular block in the PACU in children who were assessed intraoperatively using qualitative devices and/or clinical signs and who received neostigmine antagonism. This is improved with the use of sugammadex, but reliable antagonism with sugammadex in the absence of monitoring cannot be supported by our results. Thus, exclusive use of clinical signs to support recovery must be rejected as inadequate, and routine objective monitoring must be incorporated as an essential element for the safe management of neuromuscular block and its antagonism. We wholeheartedly support the movement of anesthesia societies across the world to recommend objective, quantitative monitoring in all adult and pediatric patients receiving neuromuscular blocking agents.
